# Racial/Ethnic and Regional Disparities in Opioid-Involved Overdose Deaths among Children and Adolescents in the United States

**DOI:** 10.1007/s40615-025-02554-y

**Published:** 2025-07-24

**Authors:** Greta Muriel Eikermann, Can Martin Ludeke, Annika Eyth, Aline M. Grimm, Tina Ramishvili, Felix Borngaesser, Maira Rudolph, Nicole Aber, Corinne M. Kyriacou, Giselle D. Jaconia, Jerry Chao, Ibraheem M. Karaye

**Affiliations:** 1 Ethical Culture Fieldston School, New York, New York, NY USA; 2 Department of Anesthesiology, Montefiore Medical Center, Albert Einstein College of Medicine, 111 East 210th Street, Bronx, NY 10467 USA; 3 Intensive Care, Emergency Medicine, and Pain Therapy, Klinikum Oldenburg AöR, Universitätsmedizin Oldenburg, University Clinic for Anesthesiology, Oldenburg, Germany; 4 Department for Anesthesiology and Intensive Care Medicine, University of Cologne, Faculty of Medicine and University Hospital Cologne, Cologne, Germany; 5 Department of Population Health, Hofstra University, Hempstead, NY USA

**Keywords:** Opioids, Race/ethnicity, Disparities, Children, Adolescents, Mortality, United States, Epidemic

## Abstract

**Background:**

Despite the opioid crisis being declared a national emergency in 2017, few studies have examined disparities in overdose mortality trends among children and adolescents. This study assessed trends in opioid-involved overdose mortality among U.S. individuals aged 0 to 19 years, categorized by age, sex, race/ethnicity, census region, opioid type (prescription, synthetic, and heroin), and county rural/urban designation, from 1999 to 2019.

**Methods:**

Mortality data were sourced from the Centers for Disease Control and Prevention's Wide-ranging Online Data for Epidemiologic Research Database. Opioid-related deaths were identified using ICD-10 codes. Crude and age-adjusted mortality rates (AAMR) were assessed by age, sex, race/ethnicity, census region, opioid type, and county rural/urban designation. Temporal trends were analyzed using Joinpoint regression to estimate annual percentage changes (APC) and average APC. 95% confidence intervals were derived using the Empirical Quantile method and the Parametric Method.

**Results:**

Between 1999 and 2019, 10,799 children and adolescents died from opioid overdoses (AAMR = 0.6 per 100,000; 95% CI: 0.6–0.6). From 2013–2019, overall mortality increased by 4.5% annually (95% CI: 0.91, 15.54). Mortality trends increased among Non-Hispanic Black (APC = 7.84; 95% CI: 5.12–10.56) and Hispanic individuals (APC = 5.29; 95% CI: 2.84–7.74) from 1999 to 2019, while remaining stable among Non-Hispanic White individuals from 2004 to 2019 (APC = -0.69; 95% CI: -2.09 to 0.58). Mortality rates also increased in the Northeast from 1999 to 2019 (APC = 4.23; 95% CI: 2.70–5.78) and in the West from 2015 to 2019 (APC = 21.96; 95% CI: 13.50–39.67), with a sharp increase in deaths involving synthetic opioids from 2014 to 2019 (APC = 43.37; 95% CI: 21.13–120.46).

**Conclusions:**

Opioid overdose mortality trends among US children and adolescents have increased in recent years. Contemporary rises are most pronounced among Non-Hispanic Black and Hispanic children, in the Northeastern and Western regions, and from synthetic opioids. The disparities in opioid-related deaths underscores the need for targeted interventions and continued  research to inform public health strategies.

**Supplementary Information:**

The online version contains supplementary material available at https://doi.org/10.1007/s40615-025-02554-y.

## Background

The opioid epidemic in the United States continues to evolve, disproportionately affecting certain populations, including children and adolescents [1]. In 2019, there were 49,860 recorded opioid-related deaths in the United States, accounting for 71% of all drug overdose deaths [2]. By 2020, this number had risen to 68,630, constituting 75% of all drug overdose deaths [2]. While much of the research on opioid-related mortality has focused on adults, recent evidence suggests that pediatric opioid exposure and mortality have also increased, raising concerns about racial/ethnic disparities in this crisis [3–7].

Fatal poisoning among children remains a significant and preventable public health concern [7]. Opioids are the leading cause of fatal poisoning in children under five years old [7]. Many children and adolescents continue to face exposure to these potent drugs within their homes and communities, increasing their risk of both unintentional and intentional harm [4–6]. Structural and systemic inequities, including disparities in healthcare access, prescribing practices, and socioeconomic conditions, contribute to racial/ethnic disparities in opioid-related outcomes [8–11]. For example, while Non-Hispanic White individuals have historically experienced higher opioid prescribing rates [9–11], Non-Hispanic Black and Hispanic individuals have faced greater barriers to treatment and overdose reversal interventions. [9–11]

In recent years, the opioid epidemic has shifted toward illicitly manufactured fentanyl and other synthetic opioids, leading to a rise in opioid-related mortality [12, 13]. Emerging evidence suggests that this shift has disproportionately impacted racial/ethnic minority populations, worsening existing disparities and underscoring the need for further investigation [13–15].

The Poison Prevention Packaging Act, enacted in 1970, mandated child-resistant packaging for approximately 30 medicines and hazardous household products in the US [16]. The implementation of this act led to a substantial decline in fatal poisonings in children, particularly those under 5 years old [17]. However, the mortality trends have plateaued in recent years [13, 17]. While child-resistant packaging has proven effective in reducing poisonings related to medications, it may not be as impactful in addressing poisonings from recreational substances. Moreover, opioid fatalities could be linked to malicious intent or neglect from caregivers, exemplified by a recent case in the Bronx, New York [18]. Parents or legal guardians also contribute to opioid overdosing in children, with preterm opioid exposure in infants being identified as a risk factor [19]. Prescription opioid use also elevates the risk of suicidal behavior among young people receiving pharmacologic pain management [20].

Each year, almost 5000 children under 6 years old are evaluated in emergency departments for opioid exposure [21]. Hospitalization rates associated with opioid exposure have increased by 100% across all pediatric age groups from 1997 to 2012, with rates doubling in children aged 1 to 4 years and tripling in adolescents aged 15 to 19 years [22]. The rate of pediatric intensive care unit (ICU) admission for opioid ingestions has also been on the rise since 1999 [23]. Opioids rank among the top two drugs most frequently suspected in fatal adverse drug reactions in children, including hypoxic-ischemic encephalopathy and cardio-respiratory arrest [24]. An analysis of the National Poison Data System from 2010 to 2014 revealed that nearly half of pediatric opioid exposures resulted in poisoning, with two-thirds being reported as unintentional, and over 90% reported to occur at home [25].

While previous studies have characterized pediatric opioid exposure and trends in opioid-associated hospitalizations and emergency department visits [22, 26, 27], limited research has examined trends in opioid-involved overdose deaths among children and adolescents, particularly by race/ethnicity, geographic region, and rural/urban designation. Gaither et al. [20, 28] analyzed national trends in pediatric opioid-related deaths, but their data were limited to the period from 1999 to 2016. Given the 2017 presidential declaration of the opioid crisis as an epidemic [29] (most recently renewed in 2025) [30] and the increasing prevalence of illicitly manufactured synthetic opioids, updating the literature with more recent data is paramount. Understanding contemporary trends in opioid overdose fatalities among children, and variations across demographic and geographic subgroups, is essential for informing targeted harm reduction efforts. Our study aims to assess the temporal trends in opioid overdose deaths among U.S. children and adolescents (aged 0 to 19 years) from 1999 to 2019, stratified by age, sex, race/ethnicity, census region, rural/urban classification, and opioid type, as prior research has demonstrated significant variations in opioid misuse and overdose deaths across these demographic and geographic subgroups. To account for potential disruptions related to the COVID-19 pandemic, we also analyzed trends with and without data from 2020, the latest year for which mortality data using bridged race categories are available.

## Materials and Methods

### Study Overview

This study utilizes the Centers for Disease Control and Prevention’s Wide-ranging Online Data for Epidemiologic Research (WONDER) database [31] and follows the Strengthening the Reporting of Observational Studies in Epidemiology (STROBE) guidelines [32, 33]. As WONDER is publicly available and de-identified, this study was deemed exempt from Institutional Review Board review [33].

### Study Design and Data Source

A retrospective analysis of a serial cross-sectional dataset was conducted for this study. The data, which were based on the death records of US residents, were obtained from the Multiple Cause of Death files [34, 35]. WONDER compiles death records of all US residents based on death certificates, which are aggregated at the national level by the National Center for Health Statistics [31].

For this study, we used data from 1999 to 2019 for the primary analysis and included 2020 in the sensitivity analysis. Data from 1999 to 2020 represent the most recent mortality estimates available based on bridged race categories [31]. We did not include data from 2021 to 2023, as these years report mortality estimates only for single-race categories [31], which are not directly comparable to earlier data.

### Identification of Opioid-Related Fatalities

Decedents of opioid overdose were identified using the International Statistical Classification of Diseases and Related Health Problems, 10th Revision (ICD-10) codes, X40-X44; X60-X64; X85; and Y10-Y14, from the underlying cause of death category, and T40.0-T40.4 and T40.6, from the multiple cause of death category. In line with a previous study, [28] we identified prescription opioid deaths based on the ICD-10 codes- T40.2, T40.3, and T40.6. Synthetic opioids (T40.4) were not included in the prescription opioid category because ICD-10 coding does not distinguish illicitly manufactured fentanyl from pharmaceutical fentanyl [28]. Instead, we analyzed synthetic opioids as a separate category, which may include both illicit and prescribed substances. Opium was not categorized as either prescription or illicit, and prescription-based methadone (T40.3) was included in all prescription opioid analyses. A complete list of the ICD-10 codes used in this study, including their definitions, is provided in Table S1.

### Identification of Child and Adolescent Deaths

We limited our analysis to individuals aged 0 to 19 years. To ensure sufficient death counts for reliable temporal trend estimation, we stratified by age groups 0 to 17 and 18 to 19 years. Finer stratifications (e.g., 5-year intervals) yielded insufficient counts, limiting trend analysis. This approach also distinguishes younger children from older adolescents, who may have differing risk profiles for opioid-involved deaths [36]. For each year of the study period, crude and age-adjusted mortality rates (AAMR), per 100,000 at-risk individuals, for opioid-involved deaths were abstracted by age, sex, race and ethnicity, census region, opioid type (prescription, synthetic, and heroin), and county urban–rural designation.

### Statistical Analysis

Joinpoint regression was utilized to analyze temporal trends in opioid overdose deaths. Initially assuming a linear trend throughout the study period, the method incorporates Joinpoints to signify inflections or changes in the trend [37]. A permutation test is then conducted to assess the significance of the introduced Joinpoint relative to the null model. If statistically significant, the Joinpoint is retained; otherwise, it is excluded. Additional Joinpoints are iteratively added, and the process is repeated using 4,499 Monte Carlo permutations, the default number, until the final model is established with the optimal number of Joinpoints.

Annual Percentage Changes (APCs) were estimated to evaluate temporal trends over short-term, piecewise segments. Average Annual Percentage Changes (AAPCs) assessed the overall temporal trend over the entire study period. Confidence intervals (95%) were derived using the Parametric Method, except for the final selected model, which employed the more accurate but computationally intensive Empirical Quantile Method (EQM) [38].

#### Sensitivity Analysis

To evaluate the robustness of our findings, we conducted additional analyses by extending the study period to include mortality estimates from 2020 and assessing temporal trends from 1999 to 2020. We also examined different age groupings (0–9, 10–18, and ≥ 19 years) and analyzed outcomes based on urban–rural classifications. Counties were categorized using the 2013 NCHS Urban–Rural Classification Scheme [39], and for this study, we dichotomized them intourban areas, defined as large central, large fringe, medium, and small metropolitan counties, and rural areas comprising micropolitan and noncore counties.

### Model Characteristics and Parameter Settings

We employed a modeling approach in which the log-transformed crude rate or AAMR was analyzed in relation to the year of death. Heteroscedasticity and correlated errors were assessed using the Breusch-Pagan test, which indicated constant variance, allowing for the selection of a model with uncorrelated errors. The interval type was specified as ‘annual,’ to facilitate yearly trend estimation.

Several parameters were set using default options, including the modeling method (grid search), the number of Joinpoints (0–3), the model selection method (permutation test), the overall significance level (P < 0.05), the number of permutations (4,499), and the segment ranges for the average annual percentage change (AAPC) and annual percentage change (APC). Grid search systematically evaluates potential Joinpoints to identify the best-fitting model.

Confidence intervals (95%) were estimated using the parametric method for all models except the final selected model, which used the empirical quantile method for greater accuracy. While the parametric approach was appropriate given variance homogeneity, it can yield overly conservative confidence intervals. To address this, EQM—introduced in Joinpoint version 4.2—generates multiple resampled datasets by adding resampled residuals to the original fit, estimates APC and AAPC for each iteration, and constructs τ (tau) confidence intervals from the percentiles of these resampled estimates [38]. Although computationally intensive, EQM improves coverage probabilities and offers greater robustness than traditional parametric intervals [46].

All statistical analysis was conducted using the Joinpoint Regression Program [40] and Stata 17.0 (College Station, Texas).

## Results

A total of 10,799 children and adolescents died from opioid-involved overdose between 1999 and 2019, resulting in an AAMR of 0.6 per 100,000 persons. The largest proportion of death was observed among boys (AAMR = 0.9; 95% CI: 0.8—0.9), American Indians/Alaska Natives (AAMR = 0.9; 95% CI: 0.8—1.0), and individuals aged between 18 and 19 years (AAMR = 3.9; 95% CI: 3.8—3.9) (Table 1).

**Table 1:** Demographic Characteristics of Opioid Overdose Decedents Aged 0 to 19 years, United States, 1999–2019

Variable	Opioid Overdose Death n (%)	Age Adjusted Annual Rate Per 100,000 Persons (95% CI)
Overall Population	10,799 (100.0)	0.6 (0.6—0.6)
Sex		
Female	3,028 (28.0)	0.3 (0.3—0.3)
Male	7,771 (72.0)	0.9 (0.8—0.9)
Race		
Non-Hispanic White	8,384 (77.6)	0.8 (0.8—0.8)
Non-Hispanic Black	866 (8.0)	0.3 (0.3—0.3)
Asian/Pacific Islander	98 (0.9)	0.1 (0.1—0.1)
American Indian/Alaska Native	164 (1.5)	0.9 (0.8—1.0)
Hispanic	1,252 (11.6)	0.4 (0.3—0.4)
Census Region		
Northeast	1,840 (17.0)	0.6 (0.6—0.6)
Midwest	2,367 (21.9)	0.6 (0.6—0.6)
South	4,178 (38.7)	0.6 (0.6—0.6)
West	2,414 (22.4)	0.6 (0.5—0.6)
Age (Years)		
0–17	3,879 (35.9)	0.2 (0.2—0.2)
18–19	6,920 (64.1)	3.9 (3.8—3.9)
Type		
^ a^ Prescription Opioids	7,121 (65.9)	0.4 (0.4—0.4)
Synthetic Opioids	2,386 (22.1)	0.1 (0.1—0.1)
Heroin	2,262 (20.9)	0.1 (0.1—0.1)
Manner of Death		
Unintentional	8,772 (81.2)	0.5 (0.5—0.5)
Suicide	549 (5.1)	0.0 (0.0—0.0)
Homicide	275 (2.5)	0.0 (0.0—0.0)
Undetermined	1,203 (11.1)	0.1 (0.1—0.1)

^a^Includes all codes other than those for opium (T40.0), Heroin (T40.1), and synthetic opioids (T40.4). ICD-10 Codes: T40.0-T40.4 and T40.6 (multiple cause); X40-X44; X60-X64; X85; and Y10-Y14 (underlying cause)

### Temporal Trends

**Fig. 1:**
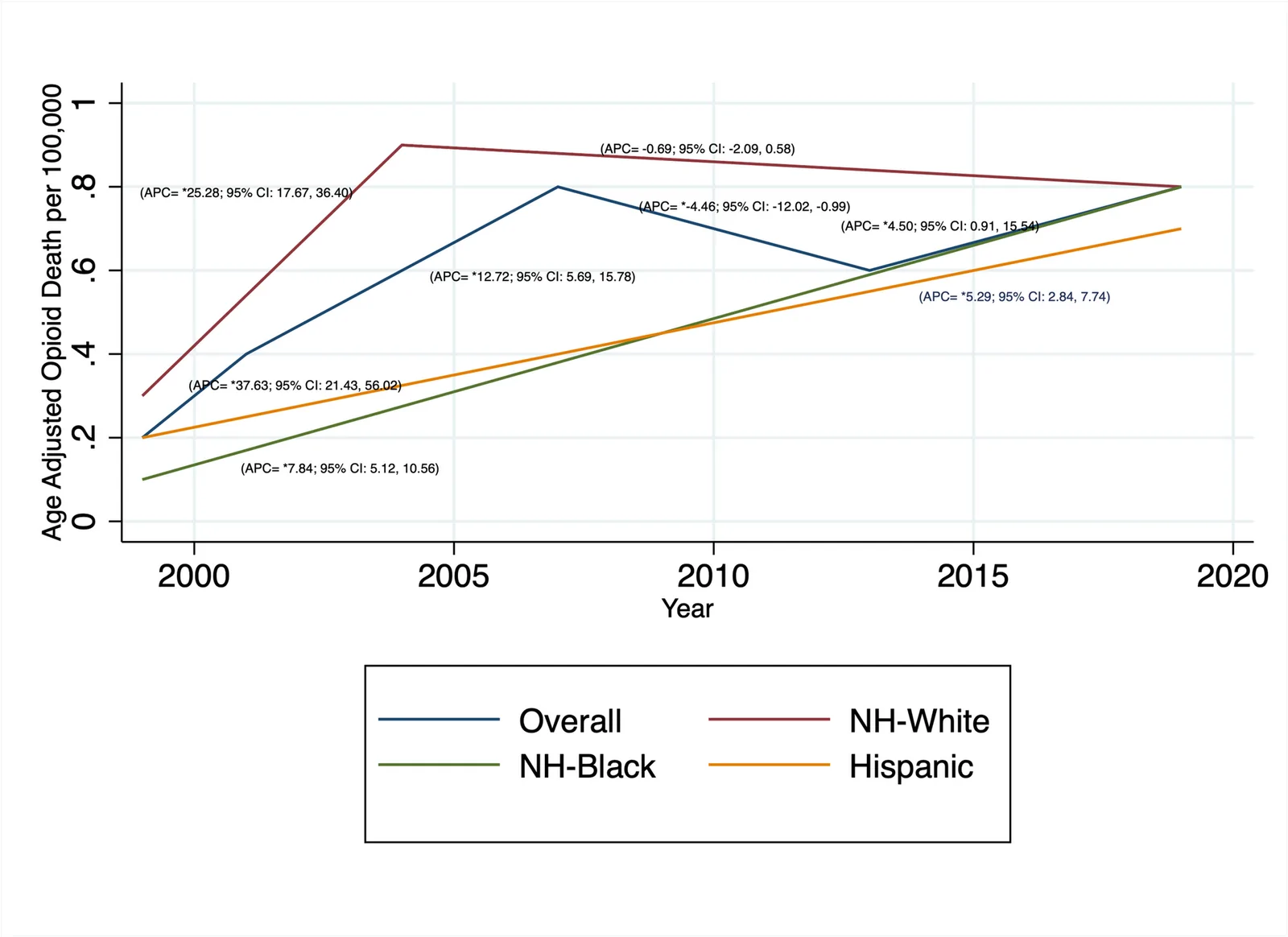
Temporal trends in opioid-involved overdose deaths among individuals aged 0 to 19 years, by race/ethnicity, United States

Overall opioid-involved overdose mortality trends increased by 37.63% per year from 1999 to 2001 (95% CI: 21.43, 56.02), and by 12.72% per year from 2001 to 2007 (95% CI: 5.69, 15.78). The trends then declined temporarily by 4.46% per year (95% CI: −12.02, −0.99), from 2007 to 2013, before increasing at an annual rate of 4.5% from 2013 to 2019 (95% CI: 0.91, 15.54) (Table 2). When stratified by race/ethnicity, the AAMR initially increased by 25.28% per year among Non-Hispanic White individuals (95% CI: 17.67, 36.40), from 1999 to 2004, and then stabilized afterwards, from 2004 to 2019 (APC = −0.69; 95% CI: −2.09, 0.58) (Table 2; Fig. 1).

**Table 2:** Annual Percentage Changes (APC) and Average Annual Percentage Changes (AAPC) in Opioid Overdose Mortality Rates among Individuals Aged 0 to 19 years, United States, 1999–2019

Variable	Trend Segment	Segment Endpoints		^c^ Crude or Age Adjusted Mortality Rate per 100,000 Persons (95% CI)	APC (95% CI)	AAPC (95% CI)
		Lower	Upper			
Overall	1	1999	2001	0.6 (0.6, 0.6)	*37.63 (21.43, 56.02)	*6.97 (6.00, 8.24)
	2	2001	2007		*12.72 (5.69, 15.78)	
	3	2007	2013		*−4.46 (−12.02, −0.99)	
	4	2013	2019		*4.50 (0.91, 15.54)	
Race/Ethnicity						
Non-Hispanic White	1	1999	2004	0.8 (0.8—0.8)	*25.28 (17.67, 36.40)	*5.25 (4.20, 6.48)
	2	2004	2019		−0.69 (−2.09, 0.58)	
Non-Hispanic Black	1	1999	2019	0.3 (0.3—0.3)	*7.84 (5.12, 10.56)	*7.84 (5.12, 10.56)
Hispanic	1	1999	2019	0.4 (0.3—0.4)	*5.29 (2.84, 7.74)	*5.29 (2.84, 7.74)
Sex						
Male	1	1999	2004	0.9 (0.8—0.9)	*21.97 (17.73, 31.31)	*5.82 (5.07, 6.75)
	2	2004	2008		6.57 (−0.51, 14.63)	
	3	2008	2013		*−6.15 (−12.41, −2.08)	
	4	2013	2019		*3.40 (0.30, 11.72)	
Female	1	1999	2003	0.3 (0.3—0.3)	*26.56 (13.42, 59.28)	*7.39 (5.74, 9.46)
	2	2003	2019		*3.06 (0.76, 4.72)	
Census Region						
Northeast	1	1999	2019	0.6 (0.6—0.6)	*4.23 (2.70, 5.78)	*4.23 (2.70, 5.78)
Midwest	1	1999	2001	0.6 (0.6—0.6)	*63.87 (26.19, 99.23)	*10.82 (8.70, 12.73)
	2	2001	2007		15.77 (−0.54, 23.19)	
	3	2007	2019		1.57 (−6.94, 5.53)	
South	1	1999	2003	0.6 (0.6—0.6)	*41.74 (19.96, 95.30)	*4.80 (2.49, 8.51)
	2	2003	2019		*−2.82 (−5.80, −0.51)	
West	1	1999	2001	0.6 (0.6—0.6)	3.34 (−8.10, 23.17)	*8.53 (7.37, 10.06)
	2	2001	2004		*33.06 (11.42, 43.93)	
	3	2004	2009		10.28 (−13.08, 14.48)	
	4	2009	2015		−9.05 (−17.94, 0.19)	
	5	2015	2019		*21.96 (13.50, 39.67)	
Age (Years)						
0–17	1	1999	2004	0.2 (0.2—0.2)	*23.18 (5.69, 91.91)	4.09 (−0.00, 8.98)
	2	2004	2019		−1.59 (−11.71, 1.25)	
18–19	1	1999	2003	3.9 (3.8—3.9)	*23.32 (19.07, 30.36)	*6.04 (5.33, 6.70)
	2	2003	2008		*7.96 (3.96, 11.69)	
	3	2008	2013		*−6.37 (−11.97, −3.93)	
	4	2013	2016		*13.75 (7.60, 18.11)	
	5	2016	2019		−3.50 (−10.66, 0.24)	
Type						
^ a^Prescription Opioid	1	1999	2007	0.4 (0.4—0.4)	*16.11 (10.54, 24.25)	0.03 (−1.52, 1.72)
	2	2007	2019		*−9.44 (−12.45, −6.91)	
^ b^Synthetic Opioid	1	2000	2003	0.1 (0.1—0.1)	*68.01 (25.44, 182.79)	*22.56 (18.22, 28.07)
	2	2003	2014		4.71 (−19.04, 9.63)	
	3	2014	2019		*43.37 (21.13, 120.46)	
Heroin	1	1999	2008	0.1 (0.1—0.1)	−0.37 (−5.35, 2.90)	−0.90 (−2.37, 0.47)
	2	2008	2016		*15.35 (10.91, 24.55)	
	3	2016	2019		*−34.95 (−46.01, −23.96)	

*Significant at *p* < 0.05; confidence interval does not include zero

^a^Includes all codes other than those for opium (T40.0), Heroin (T40.1), and synthetic opioids (T40.4). ICD-10 Codes: T40.0-T40.4 and T40.6 (multiple cause); X40-X44; X60-X64; X85; and Y10-Y14 (underlying cause)

^b^Mortality data are only available for the period, 2000 to 2020

^c^AAMRs were estimated for all variables in the study, except for'age,'for which crude rates are reported

Among Non-Hispanic Black persons, the trend increased consistently by 7.84% per year (95% CI: 95% CI: 5.12, 10.56), from 1999 to 2019. Similarly, the AAMR for Hispanic children and adolescents increased at an annual rate of 5.29% from 1999 to 2019 (95% CI: 2.84, 7.74) (Table 2; Fig. 1).

Differences were observed in AAMRs by sex, with the most recent trend increasing at an annual rate of 3.4% among males (95% CI: 0.30, 11.72), from 2013 to 2019. Among females, the AAMR initially increased at an annual rate of 26.56% from 1999 to 2003 (95% CI: 13.42, 59.28), and then at a slower rate of 3.06% from 2003 to 2019 (95% CI: 0.76, 4.72) (Table 2).

When stratified by census region, the trends increased consistently in the Northeastern region by 4.23% per year from 1999 to 2019 (95% CI: 2.70, 5.78). In the midwestern region, the trends initially increased by 63.87% annually from 1999 to 2001 (95% CI: 26.19, 99.23) and then stabilized through 2019. In the southern region, the trends increased by 41.74% per year from 1999 to 2003 (95% CI: 19.96, 95.30) and declined from 2003 to 2019 (APC = −2.82; 95% CI: −5.80, −0.51). In the western region, several fluctuations were observed, with the trends initially being stationary from 1999 to 2001 (APC = 3.34; 95% CI: −8.10, 23.17) and increased from 2001 to 2004 (APC = 33.06; 95% CI: 11.42, 43.93) and stabilized from 2004 to 2015. The most recent trend shows an increase by 21.96% per year from 2015 to 2019 (95% CI: 13.50, 39.67) (Table 2).

When stratified by age, the trend initially increased at an annual rate of 23.18% among individuals aged 0–17 years from 1999 to 2004 (95% CI: 5.69, 91.91) and stabilized afterwards from 2004 through 2019 (APC = −1.59; 95% CI: −11.71, 1.25). Among individuals aged 18 to 19 years, the most recent trend has also been stable from 2016 to 2019 (APC = −3.50; 95% CI: −10.66, 0.24) (Table 2).

Stratification by opioid type revealed differences in mortality trends. The most recent trend has declined from prescription opioids by 9.44% per year from 2007 to 2019 (95% CI: −12.45, −6.91) and from heroin, by 34.95% per year from 2016 to 2019 (95% CI: −46.01, −23.96). Conversely, for synthetic opioids, contemporary mortality trends have increased by 43.47% per year from 2014 through 2019 (95% CI: 21.13, 120.46) (Table 2).

### Sensitivity Analysis

Temporal trends in opioid-involved overdose deaths from 1999 to 2020 were directionally consistent with findings through 2019 but showed a worsening in magnitude. Mortality rates increased overall and across age groups, sexes, racial and ethnic subgroups, and U.S. census regions. Exceptions were observed by opioid type: deaths involving prescription opioids (annual percent change [APC], –7.59; 95% CI, –10.48 to –5.19) and heroin (APC, –22.70; 95% CI, –30.86 to –15.87) declined, whereas deaths involving synthetic opioids increased substantially (APC, 47.97; 95% CI, 29.94 to 91.32) (Table 3). Trends were stratified by urbanicity, revealing that rates stabilized in rural areas from 2001 to 2019, whereas in urban areas, rates increased by 5.73% annually from 2013 to 2019 (Table S2).

To identify more granular age-related trends, age was further stratified into 0–9, 10–18, and 19 years. Rates stabilized among individuals aged 0–9 years from 2013 to 2019 and among adolescents aged 10–18 years from 2007 to 2019. In contrast, among individuals aged 19 years, mortality increased by 6.04% annually from 2012 to 2019 (Table S2).

**Table 3:** Annual Percentage Changes (APC) and Average Annual Percentage Changes (AAPC) in Opioid Overdose Mortality Rates among Individuals Aged 0 to 19 years, United States, 1999–2020

Variable	Trend Segment	Segment Endpoints		APC (95% CI)	AAPC (95% CI)
		Lower	Upper		
Overall	1	1999	2005	*20.45 (13.82, 30.73)	*9.32 (7.30, 10.93)
	2	2005	2018	−0.60 (−4.30, 1.19)	
	3	2018	2020	*51.59 (16.00, 72.03)	
Race/Ethnicity					
Non-Hispanic White	1	1999	2006	*17.47 (11.68, 25.40)	*6.90 (4.95, 8.38)
	2	2006	2018	*−2.3 (−8.42, −0.04)	
	3	2018	2020	*31.77 (2.94, 49.87)	
Non-Hispanic Black	1	1999	2018	*7.25 (2.07, 9.60)	*12.56 (8.33, 14.79)
	2	2018	2020	*78.25 (11.90, 119.73)	
Hispanic	1	1999	2018	*4.60 (0.04, 7.06)	*11.03 (6.33, 13.86)
	2	2018	2020	*95.64 (13.21, 156.99)	
Sex					
Male	1	1999	2006	*17.87 (13.05, 24.50)	*9.04 (7.41, 10.42)
	2	2006	2018	*−2.31 (−5.08, −0.49)	
	3	2018	2020	*60.59 (28.94, 80.96)	
Female	1	1999	2006	*17.93 (11.32, 31.22)	*8.54 (6.88, 10.71)
	2	2006	2013	−3.04 (−18.31, 2.75)	
	3	2013	2020	*11.84 (5.66, 34.65)	
Census Region					
Northeast	1	1999	2020	*4.46 (3.13, 5.85)	*4.46 (3.13, 5.85)
Midwest	1	1999	2004	*34.13 (20.75, 63.96)	*10.40 (8.45, 12.92)
	2	2004	2020	*3.89 (1.25, 6.10)	
South	1	1999	2004	*31.76 (17.16, 57.18)	*7.89 (4.71, 10.52)
	2	2004	2018	*−4.14 (−13.46, −1.55)	
	3	2018	2020	*49.74 (2.07, 80.10)	
West	1	1999	2009	*17.03 (13.65, 20.60)	13.53 (11.84, 14.85)
	2	2009	2014	−12.31 (−22.72, 13.98)	
	3	2014	2018	10.61 (−5.80, 22.93)	
	4	2018	2020	*95.98 (57.54, 127.11)	
Age (Years)					
< 18	1	1999	2004	*24.56 (9.49, 58.92)	*9.30 (5.61, 12.53)
	2	2004	2018	−2.56 (−15.89, 0.03)	
	3	2018	2020	*76.27 (10.29, 117.49)	
8–19	1	1999	2004	*22.75 (14.11, 37.57)	*9.29 (7.25, 11.01)
	2	2004	2018	0.70 (−3.06, 2.37)	
	3	2018	2020	*44.92 (11.36, 64.81)	
Type					
^ a^Prescription Opioid	1	1999	2006	*18.87 (10.90, 32.25)	0.50 (−1.21, 2.57)
	2	2006	2020	*−7.59 (−10.48, −5.19)	
^ b^Synthetic Opioid	1	2000	2003	68.89 (27.19, 175.20)	*24.53 (20.73, 29.38)
	2	2003	2014	4.30 (−12.40, 9.52)	
	3	2014	2020	*47.97 (29.94, 91.32)	
Heroin	1	1999	2009	0.23 (−4.92, 3.35)	−0.81 (−2.35, 0.55)
	2	2009	2015	*19.99 (11.71, 39.47)	
	2	2015	2020	*−22.70 (−30.86, −15.87)	

*Significant at *p* < 0.05; confidence interval does not include zero

^a^Includes all codes other than those for opium (T40.0), Heroin (T40.1), and synthetic opioids (T40.4). ICD-10 Codes: T40.0-T40.4 and T40.6 (multiple cause); X40-X44; X60-X64; X85; and Y10-Y14 (underlying cause)

^b^Mortality data are only available for the period, 2000 to 2020

## Discussion

This national study assessed disparities in the temporal trends of opioid-involved overdose deaths among children and adolescents aged 0 to 19 years in the United States, from 1999 through 2019. The findings revealed a worsening opioid-involved mortality rate since 2013, with variations in trends across subgroups of age, sex, race/ethnicity, opioid type, rural/urban designations, and census region. Our results mirror the findings of a previous study that revealed a three-fold increase in overall opioid deaths among children and adolescents from 1999 through 2016 [28].

Although trends have recently stabilized among Non-Hispanic White individuals, they have worsened among Non-Hispanic Black and Hispanic children [12, 41]. Despite these divergent trends, Non-Hispanic White children and adolescents continue to bear the highest burden of opioid-involved mortality, as reflected in the highest age-adjusted mortality rates. This pattern parallels findings in adults, where Non-Hispanic White individuals experience the highest overall mortality, despite more recent increases among racial and ethnic minorities [14, 15, 42, 43]. The growing disparities in pediatric trends may reflect the compounded effects of structural inequities. Individuals from marginalized communities often face greater exposure to economic hardship, reduced access to high-quality health care, and educational disadvantages [44, 45]. In addition, cultural stigma surrounding mental health and limited availability of pediatric mental health and addiction services may delay intervention and increase vulnerability to substance use and overdose [46].

Previous research has documented geographic disparities in drug overdose mortality between rural and urban areas in the United States, with earlier studies (largely based on data preceding 2015) reporting a higher burden in rural counties [47–50]. However, more recent analyses suggest a reversal, with urban areas now experiencing higher increases in overdose deaths [51]. The present study supports this shift, showing that contemporary mortality trends have worsened more rapidly in urban than in rural areas. In addition to urban–rural differences, this study examined regional patterns, finding that the Northeastern and Western United States experienced the most pronounced increases in opioid-involved mortality. The drivers of these regional and urban–rural disparities remain unclear but may include differences in access to treatment and harm reduction services, the effectiveness of policy implementation, and cultural norms surrounding substance use [8, 40–43]. Understanding these contextual factors is essential to developing targeted public health strategies. Interventions such as expanded naloxone distribution and increased access to medication-assisted treatment, as advocated by Hawk et al. and McClellan et al., [52, 53] may help mitigate the rising burden and reduce geographic disparities.

The trends observed in this study align with broader national patterns, where prescription opioid-related fatalities have decreased, while deaths involving synthetic opioids have surged since 2015 [12, 13, 54]. The decline in prescription opioid-related mortality likely reflects the success of public health initiatives, including prescription monitoring programs and efforts to curb overprescribing [12]. However, the rise in synthetic opioid-related deaths underscores ongoing challenges with these potent substances. While this study did not differentiate between intentional and unintentional deaths, prior research has consistently identified accidental poisoning as the leading cause of opioid-related fatalities among children and adolescents [55–57]. Our stratification of trends by age revealed that while mortality rates have remained stable among younger children (0–9) and adolescents (10–18), the trend among individuals aged 19 has worsened significantly since 2012. This suggests that the increase in opioid-related mortality among adolescents and young adults may not be solely attributed to accidental poisoning, but could also involve intentional overdose, misused prescription opioids, or illicit drug use. Therefore, in addition to secure storage, safe disposal programs, and naloxone access, public health efforts must address the complex dynamics of opioid misuse and potential mental health factors contributing to worsening trends in older adolescents. Engaging caregivers, educators, and healthcare providers is essential to raising awareness and implementing targeted interventions that reduce harm and prevent avoidable deaths.

The sensitivity analysis extending through 2020 demonstrated a continued and more pronounced increase in opioid-involved overdose mortality among children and adolescents compared with trends from 1999 to 2019. This increase was consistent across age, sex, race and ethnicity, and census region. Although deaths involving prescription opioids and heroin declined, mortality trends attributed to synthetic opioids worsened significantly, mirroring patterns observed in the primary analysis. These findings are consistent with national trends among adults, with the CDC reporting record-high overdose deaths in 2020, largely driven by synthetic opioids [58]. The parallel increases across pediatric and adult populations suggest shared structural and social vulnerabilities, potentially exacerbated by the COVID-19 pandemic [59]. These results underscore the need for sustained and adaptive public health responses that reflect the evolving drug landscape and its impact across the lifespan.

This study has several limitations. First, the use of ecological data limits inferences at the individual level. Second, race and ethnicity classifications were based on death certificate information, which may be subject to misclassification. For example, prior studies have shown underreporting of mortality rates among Hispanic individuals when using similar sources [60]. Additionally, certain racial and ethnic groups, including American Indian/Alaska Native and Asian/Pacific Islander populations, were not included due to low and unstable death counts, which can lead to unreliable estimates. The CDC also suppresses data when death counts are fewer than 16 to protect confidentiality, further limiting subgroup analyses [61].

We relied on the publicly available CDC WONDER database rather than restricted-use files [62]. However, the public files contained all variables necessary to address our study objectives. More granular information available in the restricted files (such as exact dates of death) was not essential for our analysis. Finally, we did not examine trends within more granular intersectional subgroups (e.g., Hispanic adolescents residing in the Northeast) due to small sample sizes and associated instability in mortality estimates, which rendered such analyses unfeasible.

Despite these limitations, the current study exhibits noteworthy strengths. We employed Joinpoint regression, a validated approach for assessing temporal trends. [63, 64] This analysis identifies inflection points, recognizing that trends might not always be uniform over the entire study period [37]. Additionally, we utilized the most recent national data from the CDC to analyze temporal trends from 1999 through 2019 and 2020. Further, we disaggregated the data across regional and demographic subgroups, revealing specific groups that could be targeted for interventions.

## Conclusions

In this serial cross-sectional study, we identified worsening opioid-involved overdose deaths among individuals aged 0 to 19 years, with rising disparities among Non-Hispanic Black and Hispanic individuals, in the Northeastern and Western regions, and from synthetic opioids. Targeted public health strategies (including opioid awareness campaigns, naloxone access, childproof packaging, and caregiver support) are essential to mitigate risks. Continued surveillance and evidence-based policies are essential to protecting vulnerable pediatric populations.

## Supplementary Information

Below is the link to the electronic supplementary material.

ESM 1 (DOCX 18.2 KB)

ESM 2 (DOCX 21.4 KB)

## Data Availability

The data used in this study are publicly available from the Centers for Disease Control and Prevention (CDC) Wide-ranging Online Data for Epidemiologic Research (WONDER) Multiple Cause of Death database. The dataset can be accessed at https://wonder.cdc.gov/mcd-icd10.html.

## References

[1] Winstanley EL, Stover AN. The Impact of the Opioid Epidemic on Children and Adolescents. Clin Ther [Internet]. 2019;41:1655–62. Available from: https://www.sciencedirect.com/science/article/pii/S014929181930308X 10.1016/j.clinthera.2019.06.003 PMC7017799 31303278

[2] Opioid Overdose Deaths and Opioid Overdose Deaths as a Percent of All Drug Overdose Deaths | KFF [Internet]. [cited 2025 Apr 12]. Available from: https://www.kff.org/other/state-indicator/opioid-overdose-deaths/?currentTimeframe=0&sortModel=%7B%22colId%22:%22Location%22,%22sort%22:%22asc%22%7D

[3] Wing E, Saadat S, Bhargava R, Yun H, Chakravarthy B. Racial disparities in opioid prescriptions for fractures in the pediatric population. Am J Emerg Med. 2022;51:210–3. 34775193 10.1016/j.ajem.2021.10.017

[4] Burghardt LC, Ayers JW, Brownstein JS, Bronstein AC, Ewald MB, Bourgeois FT. Adult Prescription Drug Use and Pediatric Medication Exposures and Poisonings. Pediatrics. 2013;132:18–27. 23733792 10.1542/peds.2012-2978 PMC4074615

[5] Bond GR, Woodward RW, Ho M. The Growing Impact of Pediatric Pharmaceutical Poisoning. J Pediatr. 2012;160:265-270.e1. 21920539 10.1016/j.jpeds.2011.07.042

[6] Bailey JE, Campagna E, Dart RC. The Underrecognized Toll of Prescription Opioid Abuse on Young Children. Ann Emerg Med. 2009;53:419–24. 18774623 10.1016/j.annemergmed.2008.07.015

[7] Gaw CE, Curry AE, Osterhoudt KC, Wood JN, Corwin DJ. Characteristics of Fatal Poisonings Among Infants and Young Children in the United States. Pediatrics. 2023;151. 10.1542/peds.2022-059016 36897244

[8] Flores MW, Sharp A, Lu F, Cook BL. Examining Racial/Ethnic Differences in Patterns of Opioid Prescribing: Results from an Urban Safety-Net Healthcare System. J Racial Ethn Health Disparities. 2024;11:719–29. 36892815 10.1007/s40615-023-01555-z PMC9997438

[9] Morden NE, Chyn D, Wood A, Meara E. Racial Inequality in Prescription Opioid Receipt — Role of Individual Health Systems. N Engl J Med. 2021;385:342–51. 34289277 10.1056/NEJMsa2034159 PMC8402927

[10] Morales ME, Yong RJ. Racial and Ethnic Disparities in the Treatment of Chronic Pain. Pain Med. 2021;22:75–90. 33367911 10.1093/pm/pnaa427

[11] Hoffman KM, Trawalter S, Axt JR, Oliver MN. Racial bias in pain assessment and treatment recommendations, and false beliefs about biological differences between blacks and whites. Proc Natl Acad Sci. 2016;113:4296–301. 27044069 10.1073/pnas.1516047113 PMC4843483

[12] Ciccarone D. The triple wave epidemic: Supply and demand drivers of the US opioid overdose crisis. Int J Drug Policy. 2019;71:183–8. 30718120 10.1016/j.drugpo.2019.01.010 PMC6675668

[13] Understanding the Opioid Overdose Epidemic | Overdose Prevention | CDC [Internet]. [cited 2025 Apr 12]. Available from: https://www.cdc.gov/overdose-prevention/about/understanding-the-opioid-overdose-epidemic.html

[14] Karaye IM, Gonzalez J, Owens S, Jalal S, Sosa S, Alexander K, et al. Contemporary burden and trends of opioid-overdose mortality in New York State. Prev Med (Baltim). 2024;185. 10.1016/j.ypmed.2024.108010 38801836

[15] Karaye IM, Ludeke CM, Eikermann GM, Eyth A, Ramishvili T, Azimaraghi O, et al. Recent Trends in Opioid-Involved Overdose Deaths in New York City, 1999 to 2020. Int J Ment Health Addict. 2024;

[16] Consumer Product Safety Commission. POISON PREVENTION PACKAGING ACT Unofficial compilation for convenience only. 2. 2008 [cited 2025 Apr 12]; Available from: https://www.cpsc.gov/s3fs-public/pppa.pdf

[17] Qin A. Annual Report on Pediatric Poisoning Fatalities and Injuries. 2022 [cited 2025 Apr 12]; Available from: https://www.cpsc.gov/s3fs-public/AnnualReportonPediatricPoisoningFatalitiesandInjuries_January2022.pdf

[18] Halpert Madeline. Nursery boy’s fentanyl death provokes horror in Bronx [Internet]. BBC. 2023 [cited 2025 Apr 12]. Available from: https://www.bbc.com/news/world-us-canada-66895520

[19] Leyenaar JK, Schaefer AP, Wasserman JR, Moen EL, O’Malley AJ, Goodman DC. Infant Mortality Associated With Prenatal Opioid Exposure. JAMA Pediatr. 2021;175:706. 33843963 10.1001/jamapediatrics.2020.6364 PMC8042571

[20] Fine KL, Rickert ME, O’Reilly LM, Sujan AC, Boersma K, Chang Z, et al. Initiation of Opioid Prescription and Risk of Suicidal Behavior Among Youth and Young Adults. Pediatrics. 2022;149. 10.1542/peds.2020-049750 PMC9624202 35128560

[21] Lovegrove MC, Weidle NJ, Budnitz DS. Trends in Emergency Department Visits for Unsupervised Pediatric Medication Exposures, 2004–2013. Pediatrics. 2015;136:e821–9. 26347435 10.1542/peds.2015-2092 PMC4651433

[22] Gaither JR, Leventhal JM, Ryan SA, Camenga DR. National Trends in Hospitalizations for Opioid Poisonings Among Children and Adolescents, 1997 to 2012. JAMA Pediatr. 2016;170:1195. 27802492 10.1001/jamapediatrics.2016.2154 PMC7245935

[23] Kane JM, Colvin JD, Bartlett AH, Hall M. Opioid-Related Critical Care Resource Use in US Children’s Hospitals. Pediatrics. 2018;141. 10.1542/peds.2017-3335 29507166

[24] Carnovale C, Mahzar F, Scibelli S, Gentili M, Arzenton E, Moretti U, et al. Central nervous system-active drug abused and overdose in children: a worldwide exploratory study using the WHO pharmacovigilance database. Eur J Pediatr. 2019;178:161–72. 30374752 10.1007/s00431-018-3281-0

[25] Patel AM, Wheeler DC, Rose SR, Nadpara PA, Pakyz AL, Carroll NV. Prevalence and Characteristics of Pediatric Opioid Exposures and Poisonings in the United States. J Pediatr. 2019;206:148-155.e4. 30612813 10.1016/j.jpeds.2018.10.047

[26] Mazer-Amirshahi M, Mullins PM, Rasooly IR, van den Anker J, Pines JM. Trends in Prescription Opioid Use in Pediatric Emergency Department Patients. Pediatr Emerg Care. 2014;30:230–5. 24651218 10.1097/PEC.0000000000000102

[27] Tomaszewski DM, Arbuckle C, Yang S, Linstead E. Trends in Opioid Use in Pediatric Patients in US Emergency Departments From 2006 to 2015. JAMA Netw Open. 2018;1. 30646317 10.1001/jamanetworkopen.2018.6161 PMC6324333

[28] Gaither JR, Shabanova V, Leventhal JM. US National Trends in Pediatric Deaths From Prescription and Illicit Opioids, 1999–2016. JAMA Netw Open. 2018;1. 30646334 10.1001/jamanetworkopen.2018.6558 PMC6324338

[29] American Heart Association. President declares opioid epidemic a national public health emergency | AHA News [Internet]. 2017 [cited 2025 Apr 12]. Available from: https://www.aha.org/news/headline/2017-10-26-president-declares-opioid-epidemic-national-public-health-emergency?utm_source=chatgpt.com

[30] US Department of Health and Human Services. Secretary Kennedy Renews Public Health Emergency Declaration to Address National Opioid Crisis | HHS.gov [Internet]. 2025 [cited 2025 Apr 12]. Available from: https://www.hhs.gov/press-room/secretary-kennedy-opiod-crisis-emergency-declaration.html

[31] CDC. CDC WONDER: Multiple Cause of Death, 1999–2020 Request [Internet]. 2025 [cited 2025 Apr 13]. Available from: https://wonder.cdc.gov/mcd-icd10.html

[32] Ghaferi AA, Schwartz TA, Pawlik TM. STROBE Reporting Guidelines for Observational Studies. JAMA Surg. 2021;156:577–8. 33825815 10.1001/jamasurg.2021.0528

[33] von Elm E, Altman DG, Egger M, Pocock SJ, Gøtzsche PC, Vandenbroucke JP. Strengthening the reporting of observational studies in epidemiology (STROBE) statement: guidelines for reporting observational studies. BMJ. 2007;335:806–8. 17947786 10.1136/bmj.39335.541782.AD PMC2034723

[34] CDC WONDER: About Multiple Cause of Death, 1999–2020.

[35] CDC: Understanding the Epidemic. 2023.

[36] Chua K-P, Brummett CM, Conti RM, Bohnert A. Association of Opioid Prescribing Patterns With Prescription Opioid Overdose in Adolescents and Young Adults. JAMA Pediatr. 2020;174:141. 31841589 10.1001/jamapediatrics.2019.4878 PMC6990690

[37] Kim H-J, Fay MP, Feuer EJ, Midthune DN. Permutation tests for joinpoint regression with applications to cancer rates. Stat Med. 2000;19:335–51. 10649300 10.1002/(sici)1097-0258(20000215)19:3<335::aid-sim336>3.0.co;2-z

[38] National Cancer Institute. Empirical Quantile Confidence Interval [Internet]. 2025 [cited 2025 Apr 13]. Available from: https://surveillance.cancer.gov/help/joinpoint/setting-parameters/method-and-parameters-tab/apc-aapc-tau-confidence-intervals/empirical-quantile

[39] National Center for Health Statistics. 2013 NCHS Urban-Rural Classification Scheme for Counties Series 2, Number 166. 2013 [cited 2025 Apr 13]; Available from: https://www.cdc.gov/nchs/data/series/sr_02/sr02_166.pdf

[40] National Cancer Institute. Joinpoint Regression Program [Internet]. 2025 [cited 2025 Apr 13]. Available from: https://surveillance.cancer.gov/joinpoint/

[41] Townsend T, Kline D, Rivera-Aguirre A, Bunting AM, Mauro PM, Marshall BDL, et al. Racial/Ethnic and Geographic Trends in Combined Stimulant/Opioid Overdoses, 2007–2019. Am J Epidemiol. 2022;191:599–612. 35142341 10.1093/aje/kwab290 PMC9077116

[42] Alexander MJ, Kiang MV, Barbieri M. Trends in Black and White Opioid Mortality in the United States, 1979–2015. Epidemiology. 2018;29:707–15. 29847496 10.1097/EDE.0000000000000858 PMC6072374

[43] Kiang MV, Tsai AC, Alexander MJ, Rehkopf DH, Basu S. Racial/Ethnic Disparities in Opioid-Related Mortality in the USA, 1999–2019: the Extreme Case of Washington DC. J Urban Health. 2021;98:589–95. 34664185 10.1007/s11524-021-00573-8 PMC8566633

[44] Camacho S, Henderson SC. The Social Determinants of Adverse Childhood Experiences: An Intersectional Analysis of Place, Access to Resources, and Compounding Effects. Int J Environ Res Public Health. 2022;19:10670. 36078386 10.3390/ijerph191710670 PMC9518506

[45] Trent M, Dooley DG, Dougé J, Cavanaugh RM, Lacroix AE, Fanburg J, et al. The Impact of Racism on Child and Adolescent Health. Pediatrics. 2019;144.

[46] Amaro H, Sanchez M, Bautista T, Cox R. Social vulnerabilities for substance use: Stressors, socially toxic environments, and discrimination and racism. Neuropharmacology. 2021;188. 33716076 10.1016/j.neuropharm.2021.108518 PMC8126433

[47] Monnat SM. The contributions of socioeconomic and opioid supply factors to U.S. drug mortality rates: Urban-rural and within-rural differences. J Rural Stud. 2019;68:319–35.

[48] Pear VA, Ponicki WR, Gaidus A, Keyes KM, Martins SS, Fink DS, et al. Urban-rural variation in the socioeconomic determinants of opioid overdose. Drug Alcohol Depend. 2019;195:66–73. 30592998 10.1016/j.drugalcdep.2018.11.024 PMC6375680

[49] Keyes KM, Cerdá M, Brady JE, Havens JR, Galea S. Understanding the Rural-Urban Differences in Nonmedical Prescription Opioid Use and Abuse in the United States. Am J Public Health. 2014;104:e52–9. 24328642 10.2105/AJPH.2013.301709 PMC3935688

[50] Mack KA, Jones CM, Ballesteros MF. Illicit Drug Use, Illicit Drug Use Disorders, and Drug Overdose Deaths in Metropolitan and Nonmetropolitan Areas - United States. MMWR Surveill Summ. 2017;66:1–12. 29049278 10.15585/mmwr.ss6619a1 PMC5829955

[51] Post LA, Lundberg A, Moss CB, Brandt CA, Quan I, Han L, et al. Geographic Trends in Opioid Overdoses in the US From 1999 to 2020. JAMA Netw Open. 2022;5. 35900768 10.1001/jamanetworkopen.2022.23631 PMC9335141

[52] McClellan C, Lambdin BH, Ali MM, Mutter R, Davis CS, Wheeler E, et al. Opioid-overdose laws association with opioid use and overdose mortality. Addict Behav. 2018;86:90–5. 29610001 10.1016/j.addbeh.2018.03.014

[53] Hawk KF, Vaca FE, D’Onofrio G. Reducing Fatal Opioid Overdose: Prevention, Treatment and Harm Reduction Strategies. Yale J Biol Med. 2015;88:235–45. 26339206 PMC4553643

[54] Rudd RA, Seth P, David F, Scholl L. Increases in Drug and Opioid-Involved Overdose Deaths — United States, 2010–2015. MMWR Morb Mortal Wkly Rep. 2016;65:1445–52. 28033313 10.15585/mmwr.mm655051e1

[55] Sandelich S, Hooley G, Hsu G, Rose E, Ruttan T, Schwarz ES, et al. Acute opioid overdose in pediatric patients. J Am Coll Emerg Physicians Open. 2024;5. 38464332 10.1002/emp2.13134 PMC10920943

[56] Tadros A, Layman SM, Davis SM, Bozeman R, Davidov DM. Emergency department visits by pediatric patients for poisoning by prescription opioids. Am J Drug Alcohol Abuse. 2016;42:550–5. 27398815 10.1080/00952990.2016.1194851 PMC5055434

[57] Bishop-Freeman SC, Young KA, Aurelius MB, Hudson JS. Pediatric opioid fatalities: What can we learn for prevention? J Forensic Sci. 2021;66:1410–9. 33893645 10.1111/1556-4029.14725

[58] Hedegaard H, Miniño AM, Spencer MR, Warner M. Drug Overdose Deaths in the United States, 1999–2020. NCHS Data Brief. 2021;1–8. 34978529

[59] Gomes T, Ledlie S, Tadrous M, Mamdani M, Paterson JM, Juurlink DN. Trends in Opioid Toxicity-Related Deaths in the US Before and After the Start of the COVID-19 Pandemic, 2011–2021. JAMA Netw Open. 2023;6. 37418260 10.1001/jamanetworkopen.2023.22303 PMC10329206

[60] Arias E, Eschbach K, Schauman WS, Backlund EL, Sorlie PD. The Hispanic Mortality Advantage and Ethnic Misclassification on US Death Certificates. Am J Public Health. 2010;100:S171–7. 19762677 10.2105/AJPH.2008.135863 PMC2837441

[61] CDC. Suppression of Rates and Counts | U.S. Cancer Statistics | CDC [Internet]. 2025 [cited 2025 Apr 13]. Available from: https://www.cdc.gov/united-states-cancer-statistics/technical-notes/suppression.html

[62] CDC. Restricted-Use Vital Statistics Data [Internet]. 2025 [cited 2025 Apr 13]. Available from: https://www.cdc.gov/nchs/nvss/nvss-restricted-data.htm

[63] Barrio G, Pulido J, Bravo MJ, Lardelli-Claret P, Jiménez-Mejías E, de la Fuente L. An example of the usefulness of joinpoint trend analysis for assessing changes in traffic safety policies. Accid Anal Prev. 2015;75:292–7. 25543100 10.1016/j.aap.2014.12.010

[64] Gillis D, Edwards BPM. The utility of joinpoint regression for estimating population parameters given changes in population structure. Heliyon. 2019;5. 31768426 10.1016/j.heliyon.2019.e02515 PMC6872810

