# Supplementary material for: Racial/Ethnic and Regional Disparities in Opioid-Involved Overdose Deaths among Children and Adolescents in the United States
Source: J Racial Ethn Health Disparities. 2025 Jul 24;13(5):4020–30. doi: 10.1007/s40615-025-02554-y (PMC13546327; doi:10.1007/s40615-025-02554-y)
Supplement: Supplementary file 1 [file 40615_2025_2554_MOESM1_ESM.docx]

**Table S1.** *International Statistical Classification of Diseases and Related Health Problems, Tenth Revision* (*ICD-10*), Codes for Opioid Overdose Deaths among Children Aged 0-19 Years in the United States, 1999-2019.

| ^a^ ICD-10 Code | Description |
| --- | --- |
| Multiple Cause of Death |  |
| T40.0 | Opium |
| T40.1 | Heroin |
| T40.2 | Other opioids |
| T40.3 | Methadone |
| T40.4 | Other synthetic narcotics |
| T40.6 | Other and unspecified narcotics |
| Underlying Cause of Death |  |
| X40 | Accidental poisoning by and exposure to nonopioid analgesics, antipyretics and antirheumatics |
| X41 | Accidental poisoning by and exposure to antiepileptic, sedative-hypnotic, anti-parkinsonism and psychotropic drugs, not elsewhere classified) |
| X42 | Accidental poisoning by and exposure to narcotics and psychodysleptics [hallucinogens], not elsewhere classified |
| X43 | Accidental poisoning by and exposure to other drugs acting on the autonomic nervous system |
| X44 | Accidental poisoning by and exposure to other and unspecified drugs, medicaments and biological substances |
| X60 | Intentional self-poisoning by and exposure to nonopioid analgesics, antipyretics and antirheumatics |
| X61 | Intentional self-poisoning by and exposure to antiepileptic, sedative-hypnotic, anti-parkinsonism and psychotropic drugs, not elsewhere classified |
| X62 | Intentional self-poisoning by and exposure to narcotics and psychodysleptics [hallucinogens], not elsewhere classified |
| X63 | Intentional self-poisoning by and exposure to other drugs acting on the autonomic nervous system |
| X64 | Intentional self-poisoning by and exposure to other and unspecified drugs, medicaments and biological substances |
| X85 | Assault by drugs, medicaments and biological substances |
| Y10 | Poisoning by and exposure to nonopioid analgesics, antipyretics and antirheumatics, undetermined intent |
| Y11 | Poisoning by and exposure to antiepileptic, sedative-hypnotic, anti-parkinsonism and psychotropic drugs, not elsewhere classified, undetermined intent |
| Y12 | Poisoning by and exposure to narcotics and psychodysleptics [hallucinogens], not elsewhere classified, undetermined intent |
| Y13 | Poisoning by and exposure to other drugs acting on the autonomic nervous system, undetermined intent |
| Y14 | Poisoning by and exposure to other and unspecified drugs, medicaments and biological substances, undetermined intent |
| ^a^ ICD-10: International Statistical Classification of Diseases and Related Health Problems, 10^th^ Revision Codes | |
