# Supplementary material for: Racial/Ethnic and Regional Disparities in Opioid-Involved Overdose Deaths among Children and Adolescents in the United States
Source: J Racial Ethn Health Disparities. 2025 Jul 24;13(5):4020–30. doi: 10.1007/s40615-025-02554-y (PMC13546327; doi:10.1007/s40615-025-02554-y)
Supplement: Supplementary file 2 [file 40615_2025_2554_MOESM2_ESM.docx]

| Variable | Trend Segment | Segment Endpoints | | APC (95% CI) | AAPC (95% CI) |
| --- | --- | --- | --- | --- | --- |
|  |  | Lower | Upper |  |  |
| ^a^ Urbanization |  | | | | |
| Rural | 1 | 1999 | 2001 | *89.89 (35.54, 153.30) | *8.19 (5.62, 11.01) |
|  | 2 | 2001 | 2006 | 20.40 (-9.56, 30.38) |  |
|  | 3 | 2006 | 2019 | -4.78 (-12.82, 1.18) |  |
| Urban | 1 | 1999 | 2001 | *45.09 (25.59, 65.53) | *7.35 (6.31, 8.74) |
|  | 2 | 2001 | 2008 | *8.87 (5.18, 12.10) |  |
|  | 3 | 2008 | 2013 | *-4.98 (-12.96, -0.75) |  |
|  | 4 | 2013 | 2019 | *5.73 (2.04, 18.28) |  |
| Age (Years) |  | | | | |
| 0-9 | 1 | 1999 | 2003 | *27.43 (18.83, 42.84) | *4.86 (3.95, 6.03) |
|  | 2 | 2003 | 2019 | -0.13 (-1.16, 0.84) |  |
| 10-18 | 1 | 1999 | 2007 | *15.84 (10.98, 21.64) | *5.01 (3.62, 6.43) |
|  | 2 | 2007 | 2014 | -6.52 (-16.93, 9.82) |  |
|  | 3 | 2014 | 2019 | 5.60 (-1.68, 24.02) |  |
| 19 | 1 | 1999 | 2002 | *36.68 (16.46, 60.42) | *7.87 (3.42, 12.51) |
|  | 2 | 2002 | 2008 | 7.32 (-0.10, 15.29) |  |
|  | 3 | 2008 | 2012 | -6.23 (-20.10, 10.06) |  |
|  | 4 | 2012 | 2019 | *6.04 (1.60, 10.68) |  |
| *Significant at p<0.05; confidence interval does not include zero.  ^a^ Counties are classified based on the 2013 NCHS Urban-Rural Classification Scheme. Urban areas include large central, large fringe, medium, and small metropolitan counties. Rural areas include micropolitan and noncore counties, which are outside metropolitan statistical areas and have smaller or dispersed populations. | | | | | |

Table S2. Temporal Trends in Opioid Overdose Mortality Rates among Individuals Aged 0 to 19 years, by Urbanization and Age, United States, 1999-2019.
